# Identification of Core Gene Biomarkers in Patients with Diabetic Cardiomyopathy

**DOI:** 10.1155/2018/6025061

**Published:** 2018-12-19

**Authors:** Ning Li, Haiming Wu, Rongxin Geng, Qizhu Tang

**Affiliations:** ^1^Department of Cardiology, Renmin Hospital of Wuhan University, Cardiovascular Research Institute of Wuhan University, Hubei Key Laboratory of Cardiology, Wuhan, China; ^2^Department of Neurosurgery, Renmin Hospital of Wuhan University, China

## Abstract

Diabetic cardiomyopathy (DCM) is a disorder of the myocardium in diabetic patients, which is one of the critical complications of diabetes giving rise to an increased mortality. However, the underlying mechanisms of DCM remain incompletely understood presently. This study was designed to screen the potential molecules and pathways implicated with DCM. GSE26887 involving 5 control individuals and 7 DCM patients was selected from the GEO database to identify the differentially expressed genes (DEGs). DAVID was applied to perform gene ontology (GO) and the Kyoto Encyclopedia of Genes and Genomes (KEGG) analyses. A protein-protein interaction (PPI) network was also constructed to visualize the interactions among these DEGs. To further validate significant genes and pathways, quantitative real-time PCR (qPCR) and Western blot were performed. A total of 236 DEGs were captured, including 134 upregulated and 102 downregulated genes. GO, KEGG, and the PPI network disclosed that inflammation, immune disorders, metabolic disturbance, and mitochondrial dysfunction were significantly enriched in the development of DCM. Notably, IL6 was an upregulated hub gene with the highest connectivity degree, suggesting that it may interact with a great many molecules and pathways. Meanwhile, SOCS3 was also one of the top 15 hub genes in the PPI network. Herein, we detected the protein level of STAT3 and SOCS3 in a mouse model with DCM. Western blot results showed that the protein level of SOCS3 was significantly lower while phosphorylated-STAT3 (P-STAT3) was activated in mice with DCM. *In vitro* results also uncovered the similar alterations of SOCS3 and P-STAT3 in cardiomyocytes and cardiac fibroblasts induced by high glucose (HG). However, overexpression of SOCS3 could significantly reverse HG-induced cardiomyocyte hypertrophy and collagen synthesis of cardiac fibroblasts. Taken together, our analysis unveiled potential biomarkers and molecular mechanisms in DCM, which could be helpful to the diagnosis and treatment of DCM.

## 1. Introduction

Diabetic cardiomyopathy (DCM) is a common cardiac dysfunction which affects approximately 12% of diabetic patients, giving rise to overtly higher cardiovascular morbidity and mortality than those without glycemia [1]. DCM is featured by ventricular diastolic and (or) systolic dysfunction occurring in patients with type 1 or type 2 diabetes independent of hypertension, coronary artery disease (CAD), and other cardiovascular diseases [2]. The pathogenesis of DCM is a multistep process, which is implicated with the alterations of various vital events, including mitochondrial dysfunction, altered lipid metabolism, endoplasmic reticulum stress, oxidative stress, inflammation, and epigenetic changes [3, 4]. Evidence is mounting that the occurrence and progression of DCM are triggered by the abnormal expression or mutation of genes, such as S6 kinase 1 (S6K1) [5], CD36 [6], peroxisome proliferator-activated receptor-*α* (PPAR-*α*) [7], and protein kinase C (PKC) [8]. Currently, the diagnosis of DCM in clinics mainly relies on the serum natriuretic peptide (NAPP) level and other noninvasive tests involving electrocardiography, to clarify ventricular overload; X-ray, to evaluate fluid accumulation; and conventional cardiac ultrasound, to figure out cardiac structure and function. However, these methods for DCM diagnosis lack the specificity and efficiency; it was thus difficult for us to obtain early and accurate diagnosis as well as treatment, so some patients with DCM missed the best opportunity for treatment, thereby increasing death risk [1]. Hence, identifying the specific and sensitive genes or proteins which can help us confirm the patients with DCM as early as possible is of vital significance, not only for more accurate diagnosis, better treatment, and ideal prognosis but also for an overall understanding of the molecular mechanisms underlying DCM.

Bioinformatic analysis and gene expression profiling analysis have enabled us to screen molecular markers among healthy individuals and patients, which provides novel insights into diseases at multiple levels ranging from the alterations of copy number at the genome level to gene expression at transcriptome level, and even epigenetic alterations. However, in fact, the application of these microarrays has not gained popularity as expected in clinics because of an overwhelming amount of genes identified by gene profiling, lack of validation or repeatability, and intricate statistical analyses [9–11]. Therefore, for the purpose of putting these expression profiles in clinical practice as quickly as possible, it is of necessity to validate a suitable amount of genes and develop a proper and ideal approach which could be operated routinely.

In the current study, the gene expression profile of GSE26887 was downloaded from the Gene Expression Omnibus (GEO, http://www.ncbi.nlm.nih.gov/geo/) and analyzed using the GEO2R online tool to comprehensively identify the differentially expressed genes (DEGs) between DCM and healthy individuals. Furthermore, we also analyzed the gene ontology (GO) involving biological process (BP), molecular function (MF), cellular component (CC), and KEGG pathways of these DEGs. Subsequently, we carried out a protein-protein interaction (PPI) network of these DEGs and chose the top 15 hub genes with a high degree of connectivity. Meanwhile, we also re-identified the top 15 hub genes by PCR and Western blot.

## 2. Materials and Methods

### 2.1. Microarray Data

We obtained the microarray of GSE26887 from the National Center for Biotechnology Information (NCBI) GEO database, which is a free and publicly available database [10]. The GSE26887 dataset possesses 24 samples in all, containing 5 normal individuals, 7 patients with diabetic cardiomyopathy, and 12 nondiabetic-heart failure patients with ischemic cardiomyopathy, which was based on the GPL6244 platform [HuGene-1_0-st] Affymetrix Human Gene 1.0 ST Array [transcript (gene) version] by Greco et al. We also downloaded the Series Matrix File of GSE26887 from the GEO database in Pubmed. All of these cardiac tissues were acquired from the vital, noninfarcted zone derived from patients with dilated hypokinetic postischemic cardiomyopathy in surgical ventricular restoration. Inclusion criteria of the diabetic group for this microarray were blood glucose ≥126 mg/dL, previous type 2 diabetes mellitus (T2DM) diagnosis, or receiving antidiabetic therapy, and those for the nondiabetic group were blood glucose <100 mg/dL and HbA1c n.v. 4.8–6.0%. Additionally, heart failure patients were matched for ejection fraction (LVEF), end systolic volume (ESV), sex, age, smoke habits, ethnic distribution, body mass index (BMI), hypertension, and glomerular filtration rate. The gene expression profile was assessed by Affymetrix GeneChip Human Gene 1.0 ST array using total RNA extracted from the above samples.

### 2.2. Identification of DEGs

We screened the DEGs between DCM and healthy samples using GEO2R (http://www.ncbi.nlm.nih.gov/geo/geo2r), an interactive analysis tool for the GEO database on the basis of R language. Consistent with the previous criteria [12, 13], we defined the genes with logFC < −1 (downregulated genes) or logFC > 1 (upregulated genes) as differentially expressed. Meanwhile, the adjusted *P* value < 0.05 was regarded statistically different, aiming at reducing the false positive rate. Furthermore, after downloading the relatively raw TXT data, we also applied visual hierarchical cluster analysis to display the volcano plot and heat map of DCM and healthy samples using ImageGP (http://www.ehbio.com/ImageGP/index.php/Home/Index/index.html).

### 2.3. Protein-Protein Interaction (PPI) Network

The PPI network could identify the core hub genes and key gene modules between healthy individuals and patients [14]. Firstly, we used the Search Tool for the Retrieval of Interacting Genes/Proteins (STRING), which is a well-known database containing the predicted and recognized protein interactions (https://string-db.org/), to identify the PPI association. Subsequently, we applied Cytoscape software platform on the basis of the PPI associations to construct the PPI network. Top 15 hub genes were selected according to the ranking order of connectivity degree.

### 2.4. Gene Ontology (GO) and Kyoto Encyclopedia of Genes and Genomes (KEGG) Pathway Analysis

GO analysis can annotate a collection of genes with functions involving molecular function (MF), cellular components (CC), and biological process (BP) [15]. The KEGG pathway is a group of databases which could hint biological pathways of certain genes implicated with diseases and drugs. KEGG in essence is a resource for us to receive an integrated understanding of biological functions and even some advanced genome information [16]. The GO and KEEG analysis in our study was performed by the Database for Annotation, Visualization and Integrated Discovery (DAVID, http://david.ncifcrf.gov) (version 6.7), an online biological function database integrating considerable biological data and analysis tools [17]. *P* < 0.05 should be the cut-off criterion. We also used ImageGP to construct the enrichment plots, aiming to visualize the BP, MF, CC, and KEGG pathways of these DEGs.

### 2.5. Animals

All animal experimental procedures in this study were approved by the Animal Care and Use Committee of Renmin Hospital of Wuhan University and were performed in accordance with the Care and Use of Laboratory Animals published by the US National Institute of Health (Revised 2011). Both male type 2 diabetic (db/db) (*n* = 8) and WT mice (*n* = 8) (8–10 weeks) weighing 25.2 ± 2 g were purchased from the Institute of Laboratory Animal Science, Chinese Academy of Medical Sciences (Beijing, China).

### 2.6. Quantitative Real-Time PCR (qPCR)

The mice were sacrificed by injecting excessive sodium pentobarbital. Whereafter, the left ventricles of mice were collected for further RNA detection. Total RNA was isolated using the TRIzol (Invitrogen, Carlsbad, CA, USA) assay, the concentrations and purities of which were quantified using an ultraviolet spectrophotometer. The RNA was then reversely transcribed according to the previous description [18]. The expression levels of top 5 upregulated genes and top 5 downregulated genes were normalized to GAPDH. Relative mRNA expression levels were analyzed by the 2^−∆∆^ cycle threshold (CT) method. The primer sequences are displayed in Table S1.

### 2.7. Western Blot

Protein extraction, SDS-PAGE, and immunodetection of the cardiac tissues were all performed according to our previous research. Protein expression levels were normalized to the matched total proteins or GAPDH [19].

### 2.8. Cell Culture and Treatment

Neonatal rat cardiomyocytes and neonatal rat cardiac fibroblasts were isolated according to the previous study [20]. Cardiomyocyte hypertrophy was evaluated by anti-*α*-actinin immunofluorescence staining while the phenotypic change of cardiac fibroblasts was evaluated by anti-*α*-SMA immunofluorescence staining. For cell transfection, replication-defective adenoviral vectors were employed to upregulate the expression of SOCS3. After infection, cardiomyocytes and cardiac fibroblasts were incubated with a high-glucose concentration (33 mM glucose) while the normal group was exposed to a normal glucose concentration (5.5 mM glucose).

### 2.9. Statistical Analysis

The obtained data were presented as mean ± SD (standard deviation) and assessed by the two-tailed Student's *t*-test. A difference of *P* < 0.05 was considered statistically significant.

## 3. Results

### 3.1. Identification of DEGs

**Figure 1:**
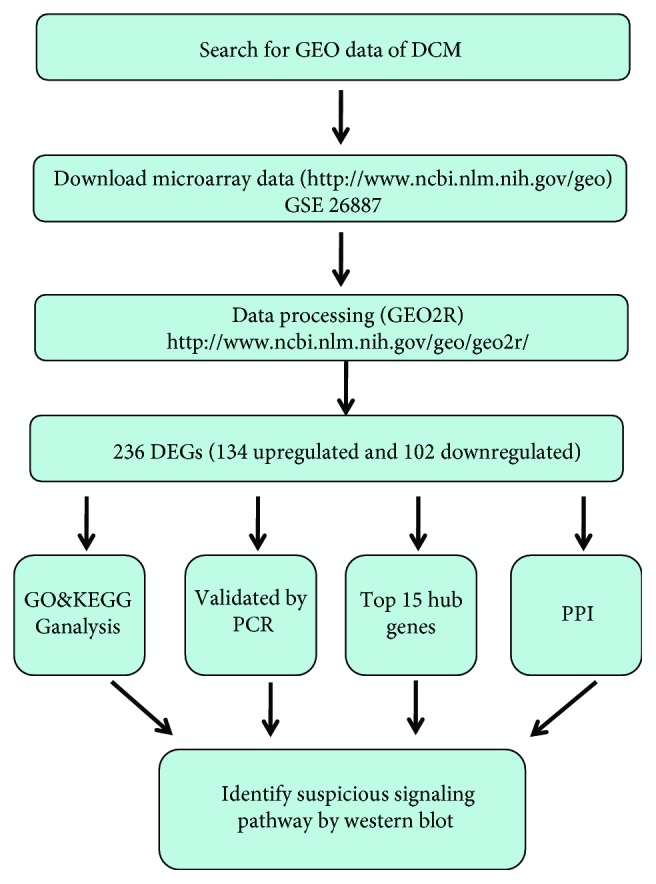
Flow diagram of the analysis procedure: data collection, preprocessing, analysis, and validation.

**Figure 2:**
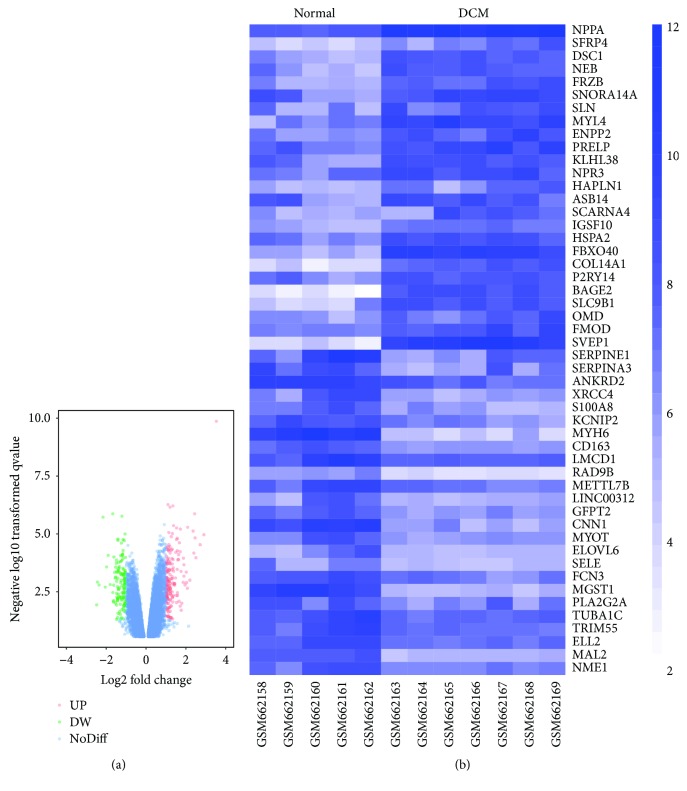
Volcano plot and heat map of the differentially expressed genes (DEGs) between normal samples and patients with diabetic cardiomyopathy (DCM). (a) Volcano plot of genes detected in DCM. Green means downregulated DEGs; red means upregulated DEGs; blue means no difference. (b) Heat map of top 25 upregulated DEGs and top 25 downregulated DEGs.

The overall flow diagram of our study is presented in Figure 1. In this study, a total of 5 normal individuals and 7 patients with DCM were analyzed. We applied the GEO2R online analysis tool with default parameters to screen the DEGs, using adjusted *P* value < 0.05 and logFC ≤ −1 or logFC ≥ 1 as the cut-off criteria. After analyzing GSE26887, 236 DEGs were captured, including 134 upregulated genes and 102 downregulated genes. The expression proportion of these DEGs is displayed in the volcano plot (Figure 2(a)). The heat map represented the top 25 upregulated genes and top 25 downregulated genes between patients with DCM and healthy individuals (Figure 2(b)). Among these 236 DEGs, the top 5 upregulated genes involved NPPA, SFRP4, DSC1, NEB, and FRZB while the top 5 downregulated genes were SERPINE1, SERPINA3, ANKRD2, XRCC4, and S100A8. The gene tiles and biological functions of these 10 genes are displayed in Table 1.

**Figure 3:**
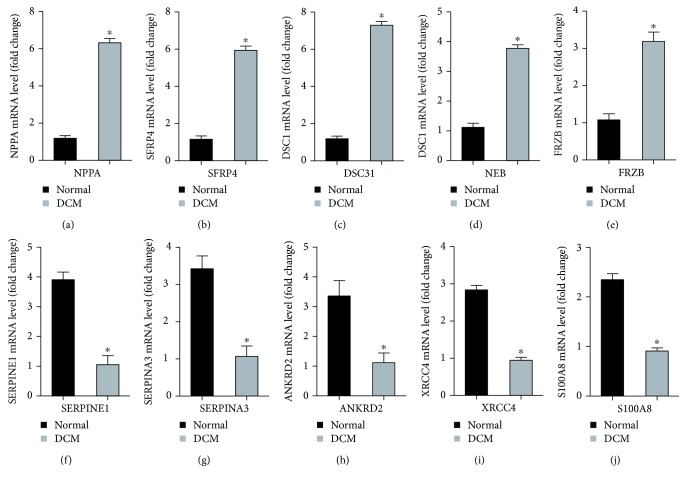
Validation of top 5 upregulated and top 5 downregulated DEGs in the mouse model of DCM. (a–e) NPPA, SFRP4, DSC31, NEB, and FRZB were significantly upregulated in the DCM group. (f–j) SERPINE1, SERPINA3, ANKRD2, ANKRD2, and S100A8 were significantly downregulated in the DCM group. ^∗^ *P* < 0.05 versus normal group.

To ensure the credibility of the microarray of GSE26887 and obtain further credible analysis, we re-identified the top 5 upregulated genes and top 5 downregulated genes via qPCR in vivo and in vitro. The results of echocardiography, hematoxylin and eosin (H&E) staining, and picrosirius red (PSR) indicated that the DCM model of db/db mice was constructed successfully (Figure S1A–B). The results from PCR demonstrated that the mRNA expression levels of NPPA, SFRP4, DSC1, NEB, and FRZB were significantly higher in the DCM group compared to the healthy group while the mRNA expression levels of SERPINE1, SERPINA3, ANKRD2, XRCC4, and S100A8 in the DCM group were statistically lower than those in the healthy group (Figures 3(a)–3(j)). Also, we detected the expression levels of these genes in cardiomyocytes and cardiac fibroblasts, respectively. In cardiac fibroblasts, the alterations of the ten genes were consistent with the mouse model (Figure S2A–B). Intriguingly, the expression level of ANKRD2 in cardiomyocytes displayed no significant difference between the normal group and the HG group. In spite of this, other nine genes in cardiomyocytes had a similar variation trend with the mouse model and cardiac fibroblasts (Figure S2C–D). One the one hand, these results increased the credibility of this microarray. On the other hand, these DEGs with the most significant difference may be the promising candidates in clinics to diagnose DCM.

### 3.2. GO Enrichment Analysis

**Figure 4:**
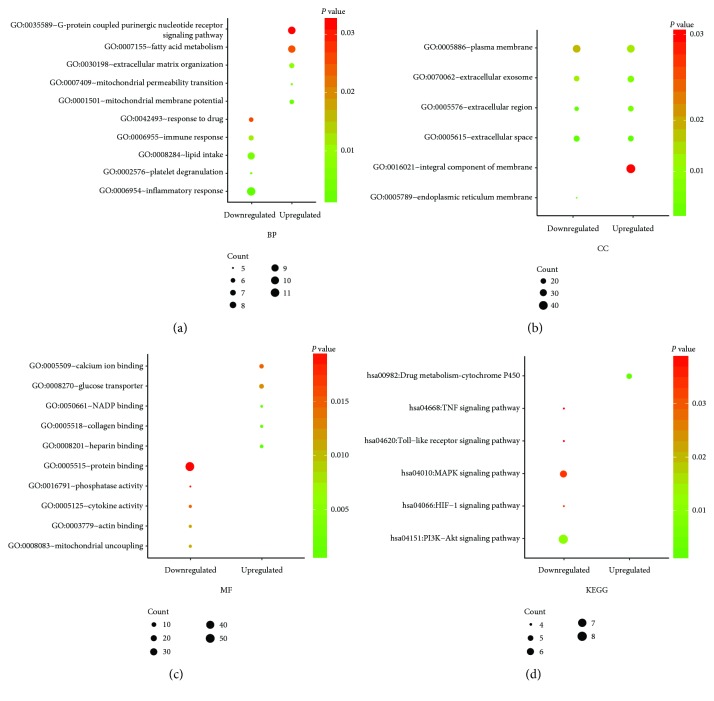
Gene ontology (GO) and Kyoto Encyclopedia of Genes and Genomes (KEGG) pathway analysis of DCM. (a) The enriched GO terms in the biological process (BP); (b) the enriched GO terms in the cellular component (CC); (c) the enriched GO terms in the molecular function (MF); (d) the enriched KEGG pathway in DCM.

The results from GO term enrichment analysis varied from expression alterations and GO classification of these DEGs. By analyzing GO enrichment of these upregulated DEGs and downregulated DEGs via DAVID, we discovered that the upregulated DEGs in BP were mainly enriched in the G-protein-coupled purinergic nucleotide receptor signaling pathway, fatty acid metabolism, mitochondrial membrane potential, extracellular matrix organization, and mitochondrial permeability transition while the downregulated DEGs in BP were enriched in inflammatory response, lipid intake, response to drug, immune response, and platelet degranulation. As for CC, the upregulated DEGs were principally enriched in the integral component of the membrane, plasma membrane, extracellular exosome, extracellular space, and extracellular region while the downregulated DEGs were enriched in the plasma membrane, extracellular space, extracellular region, extracellular exosome, and endoplasmic reticulum membrane. Additionally, MF analysis uncovered that the upregulated DEGs were principally enriched in zinc ion binding, calcium ion binding, heparin binding, collagen binding, and NADP binding. The downregulated genes were responsible for protein binding, mitochondrial uncoupling, cytokine activity, actin binding, and phosphatase activity (Table 2 and Figures 4(a)–4(c)).

### 3.3. KEGG Pathway Analysis

To acquire more comprehensive information regarding the vital pathways of those selected DEGs, KEGG pathways were also analyzed via DAVID. The results in Table 3 and Figure 4(d) unveiled the most important KEGG pathway of the downregulated and upregulated DEGs. The downregulated DEGs were mainly enriched in the PI3K-Akt signaling pathway, MAPK signaling pathway, HIF-1 signaling pathway, TNF signaling pathway, and Toll-like receptor signaling pathway. By contrast, the upregulated DEGs, namely, FMO4, FMO2, FMO3, ADH1B, and UGT2B4, had a strong correlation with drug metabolism-cytochrome P450.

### 3.4. PPI Analysis

**Figure 5:**
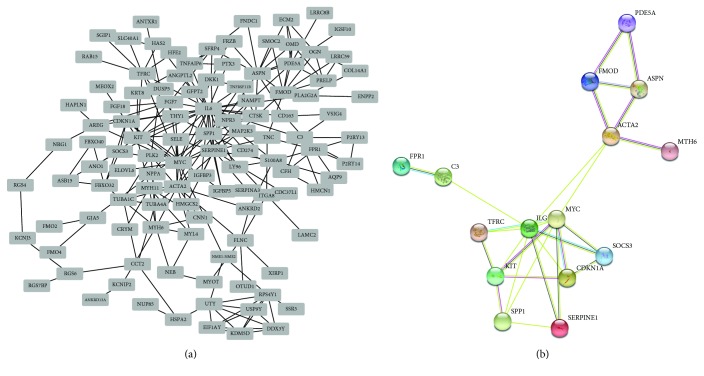
Protein-protein interaction (PPI) network. (a) The PPI network of overall DEGs and (b) the PPI network of top 15 hub genes with high connectivity degree.

Applying the STRING online tool, 120 nodes with 162 PPI relationships were identified, which accounted for approximately 90.3% of these selected DEGs (Figure 5(a)). Based on the degree of connectivity, we constructed the PPI network and selected the top 15 hub genes (Table 4). The top 15 hub genes, possessing high degree of connectivity in DCM, are as follows: IL6, MYC, ACTA2, SERPINE1, ASPN, SPP1, KIT, TFRC, FMOD, PDE5A, MYH6, FPR1, C3, CDKN1A, and SOCS3. Among these 15 hub genes, IL6, MYC, SERPINE1, SPP1, TFRC, MYH6, FPR1, C3, CDKN1A, and SOCS3 were significantly downregulated while ACTA2, ASPN, KIT, FMOD, and PDE5A were upregulated. The 15 hub genes could interact with 189 genes directly, and IL6 acted as the most intensive gene which could interact with 32 downregulated genes and 15 upregulated genes. Intriguingly, among these, hub genes also displayed strong interactions (Figure 5(b)). For instance, ACTA2 could directly interact with multiple genes (FMOD, IL6, MYH6, MYC, and ASPN), and SPP1 interacted with 4 hub genes (KIT, IL6, MYC, and SERPINE1). The details of the interactions among these 15 hub genes are shown in Table 5. Taken together, these results suggested that these hub genes, especially IL6, ACTA2 as well as SPP1 may exert critical effects in DCM.

### 3.5. Functional Analysis

**Figure 6:**
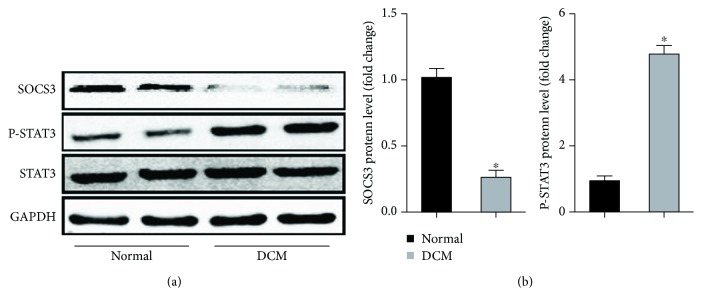
Identification of the STAT3/SOCS3 pathway in an in vivo model of DCM. SOCS3: phosphorylated-STAT3 (P-STAT3) and total STAT3 protein levels as shown by Western blot analysis.

**Figure 7:**
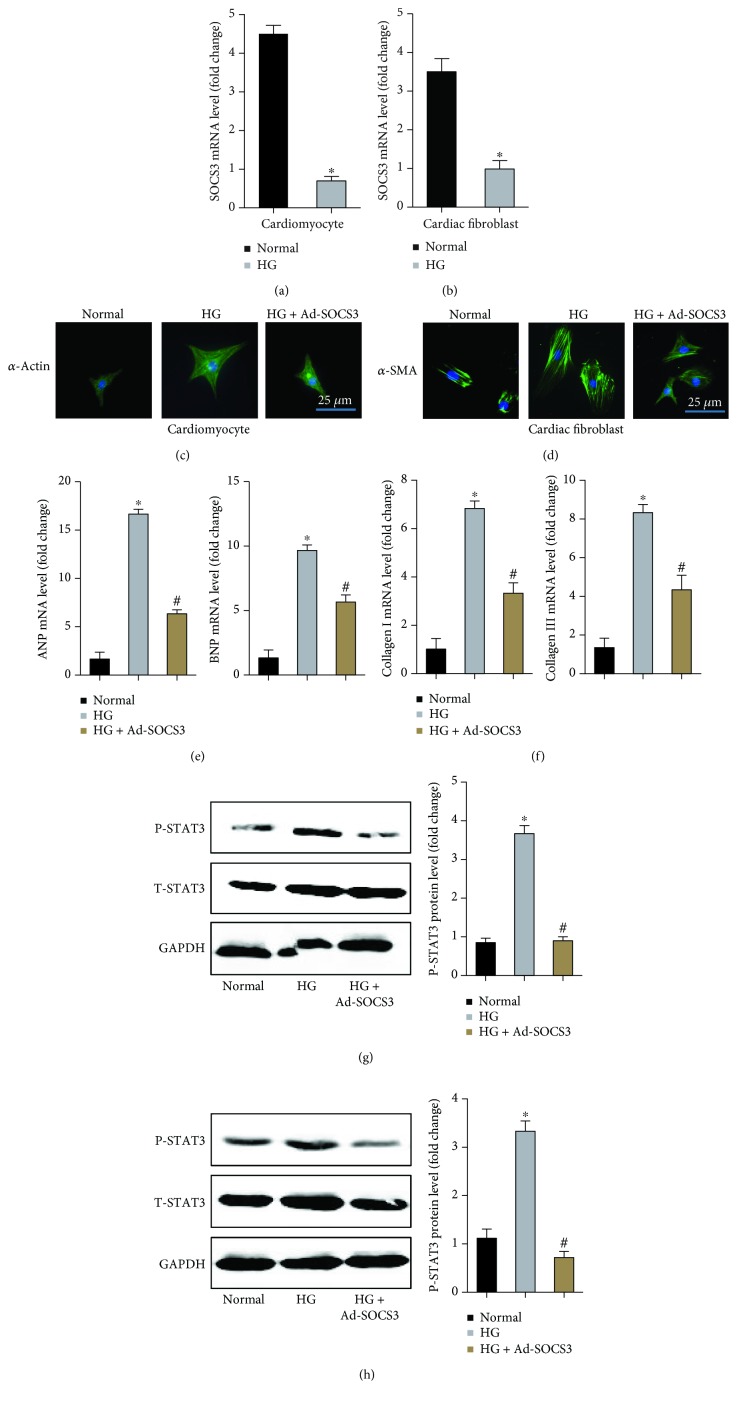
The expression and effects of SOCS3 in cardiomyocytes and cardiac fibroblasts. (a) The mRNA level of SOCS3 in cardiomyocytes induced by HG. (b) The mRNA level of SOCS3 in cardiac fibroblasts induced by HG. (c) The immunofluorescence staining of *α*-actin in cardiomyocytes induced by HG with or without SOCS3 overexpression. (d) The immunofluorescence staining of *α*-SMA in cardiac fibroblasts induced by HG with or without SOCS3 overexpression. (e) The mRNA levels of ANP and BNP in cardiomyocytes induced by HG with or without SOCS3 overexpression. (f) The mRNA levels of collagen I and collagen III in cardiac fibroblasts induced by HG with or without SOCS3 overexpression. (g, h) The protein expression of P-STAT3 and T-STAT3 in the indicated groups. ^∗^ *P* < 0.05 versus normal group and ^#^ *P* < 0.05 versus HG group.

To figure out the role of the IL-6/STAT3/SOCS3 signaling pathway in the development of DCM, we detected the protein expression levels of SCOS3, phosphorylated-STAT3 (P-STAT3), and total STAT3 between the normal group and the db/db group. The results showed that P-STAT3 had a significantly higher expression level in the DCM group compared to the normal group. Meanwhile, the level of SCOS3 was significantly downregulated in the DCM group (Figure 6). To further explore the role of the IL-6/STAT3/SOCS3 signaling pathway in cardiomyocytes and cardiac fibroblasts stimulated by HG, we firstly detected the mRNA expression of SOCS3 in cardiomyocytes and cardiac fibroblasts. As expected, the levels of SOCS3 in both cardiomyocytes and cardiac fibroblasts were significantly lower in the HG group compared with the normal group (Figures 7(a) and 7(b)). Additionally, immunofluorescent staining showed that the hypertrophic reactions of cardiomyocytes and phenotypic switching of cardiac fibroblasts were significantly abolished after SOCS3 was upregulated (Figures 7(c) and 7(d)). Meantime, hypertrophic markers and fibrotic markers were also decreased by the overexpression of SOCS3, evidenced by the lower levels of ANP, BNP, collagen I, and collagen III (Figures 7(e) and 7(f)). Western blot showed that the overexpression of SOCS3 could inhibit the phosphorylation of STAT3 (Figures 7(g) and 7(h)).

## 4. Discussion

Diabetes mellitus has been broadly regarded as one of the leading causes of morbidity and mortality for several decades worldwide. According to estimates, by 2030, there will be approximately 450 million persons with diabetes. DCM serves as the major etiological factor and death cause of patients with diabetes, the incidence of which has increased over recent years [21]. However, currently, there is no specific and efficient diagnostic methodology and treatment strategy for DCM, which is partially because of the complicated molecular mechanisms, as well as its being asymptomatic for the first several years [2]. Hence, some key diagnostic biomarkers and therapeutic targets in plasma and myocardial biopsy should be verified as early as possible. Although myocardial biopsy is not as routine as that in tumors, it does not mean that it makes no sense to perform myocardial biopsy. Takeishi and Yoshihisa retrospectively analyzed 378 patients with suspected cardiomyopathy who underwent myocardial biopsy and found that the diagnostic impact of myocardial biopsy may be relatively high in patients with suspected hypertrophic cardiomyopathy compared to those with suspected dilated cardiomyopathy [22]. Additionally, in patients with arrhythmogenic right ventricular cardiomyopathy (ARVC), Yoshida et al. detected the expression of plakoglobin and connexin 43 in myocardial biopsy specimens and confirmed the correlations between the levels of these 2 proteins and the development of ARVC, indicating that plakoglobin and connexin 43 are two specific biomarkers of arrhythmic events in ARVC [23]. Furthermore, the combination of cardiac magnetic resonance (CMR) imaging and myocardial biopsy may also improve the diagnostic value in the evaluation of cardiomyopathic conditions [24]. The above studies further supported the potential of myocardial biopsy in diagnosis of DCM. In this study, we firstly performed a comprehensive investigation on expression profiling of myocardial tissue obtained from patients with DCM. Our study included 5 normal individuals and 7 patients with DCM from the GEO database of GSE26887. In our analysis, a total of 236 DEGs (accounting for 2.6% of all genes) were found, involving 134 upregulated genes and 102 downregulated genes. By further annotating and analyzing these DEGs, we identified 10 sensitive biomarkers and top 15 hub genes among these DEGs. Additionally, we also speculated the putative mechanisms of SOCS3 contributing to DCM by Western blot.

### 4.1. The Production of IL6 Is Essential for the Development of DCM

IL6 is a critical cytokine exerting multiple physiological effects in inflammation and immune regulation, which could be secreted by a range of cell types including monocytes, mast cells, lymphocytes, macrophages, endothelial cells, keratinocytes, tumor cell lines, and fibroblasts [25]. In the innate immune system and adaptive immunity, IL6 stimulation could trigger different biological activities [26]. For example, in innate immunity, IL6 could accelerate the production of neutrophils as well as the synthesis of acute-phase proteins, thus giving rise to acute-phase response while in adaptive immunity, IL6 stimulation could increase the proliferation of B cells [27]. Notably, IL-6 pretreatment increased collagen production in cultured cardiac fibroblasts and promote interstitial fibrosis in Ang II-induced rat heart [28, 29]. Zhang et al. demonstrated that deletion of IL-6 preserved cardiac function and mitigated the interstitial fibrosis in streptozotocin-induced diabetic mice, the mechanism of which may involve the inhibitory effects of IL-6 on TGF*β*1 and miR-29 pathway [30]. Clinical trials also disclosed a strong correlation between elevated levels of circulating IL6 and heart failure severity and mortality in patients [31]. In our study, IL6 was an upregulated hub gene with the highest connectivity degree, indicating that IL6 may exert a core and predominant role in the development of DCM. Additionally, according to KEGG analysis, IL6 was significantly enriched in the PI3K/Akt signaling pathway, hypoxia-inducible factor-1 (HIF-1) signaling pathway, TNF signaling pathway, and Toll-like receptor signaling pathway. The previous study has demonstrated that HIF-1 deregulation during the early stage of diabetes gave rise to the development of DCM [32]. In diabetic retinopathy, the expression of proinflammatory IL6 and TNF-*α* were significantly inhibited after decreasing the expression of HIF-1 [33]. Hence, whether IL6 could be effectively suppressed via blocking HIF-1 in DCM, eventually alleviating inflammation in myocardium, needs to be further explored.

### 4.2. Inflammation and Immune Disorders Are Vital Pathophysiological Alterations of DCM

Another major finding of our study is that the inflammation and immune disorders mediated by cytokines may exert an important role in DCM [34]. Cardiac inflammation is one of the important features in heart failure. Enhanced proinflammatory cytokine expression levels and intensified immune cell infiltration, including macrophages and cytotoxic T lymphocytes, have been previously observed in the inflamed heart in DCM [35]. The activation of certain molecular pathways including c-Jun NH2-terminal kinase, NF-*κ*B, and p38-MAPK could aggravate the development of inflammation which displayed a strong relationship with insulin resistance, thus inducing DCM [36–38]. In the diabetic hearts of type 2 diabetes patients, elevated inflammatory cytokines, such as IL6, TNF-*α*, cell adhesion molecules, including vascular cell adhesion molecule-1 (VCAM-1) and intracellular adhesion molecule-1 (ICAM-1), and acute phase reactants, such as C-reactive protein and other inflammatory markers, have been verified [39, 40]. On the other hand, immune disorder in cardiovascular diseases has been also studied for decades. Particularly, activation of the immune system is not independent of inflammation in the progression of heart failure. In chronic heart failure, activating the immune system can usually contribute to the activation of the complement system, secretion of proinflammatory cytokines, and production and release of autoantibodies [41]. As for DCM, impaired systolic and diastolic LV function in the streptozotocin-induced diabetes, to a great extent, was correlated with increased immune cell invasion and adverse cardiac remodeling [42]. Toll-like receptors (TLRs) are a type of membrane-anchored proteins existing in various cell types involving immune cells (macrophages and lymphocytes) and nonimmune cells (cardiomyocytes) [43, 44]. Cardiac TLRs and inflammasome could interact with each other, inducing inflammation through reactive oxygen species overproduction and NF-*κ*B activation [45]. In our study, the enriched Toll-like receptor signaling pathway was observed from KEGG analysis, indicating the vital role of immunity in DCM. Meanwhile, GO analysis unveiled that the downregulated DEGs were mainly enriched in inflammatory response and immune response. Additionally, we found that the upregulated DEGs were significantly enriched in the G-protein-coupled purinergic nucleotide receptor signaling pathway in BP. To our knowledge, the nucleotides, the fundamental subunits of nucleic acids, are released by mast cells, macrophages, T cells, endothelial cells, and platelets in response to physiological activation. The purinergic nucleotide receptor could inhibit the activation of effector T cells in many allergic diseases [46]. Thus, we hypothesize that balancing the immune homeostasis by regulating the nucleotide receptor signaling pathway may be promised to be a novel strategy to treat DCM. Last but not least, many of the hub genes and top 5 downregulated or upregulated genes were also implicated with inflammation and immune response: SERPINA3 (immune response to elevated platelet cytosolic Ca^2+^), S100A8 (regulating inflammation and oxidative stress, activatingTLR4 signaling), ANKRD2 (a modulator of NF-*κ*B-mediated inflammatory), FPR1 (G protein-coupled receptor, inflammation), C3 (immune response), CDKN1A (inflammatory response gene), SOCS3 (regulating interleukin), and IL6 (proinflammatory cytokine).

Taken together, our results, in the perspective of bioinformatics, provide compelling evidence for the fact that inflammation and immune effects play critical roles in the development in DCM. Sincerely, we hope that these findings could provide new strategies and insights to identify the pivotal targets and pathways with regards to the immunologic mechanisms of DCM in future research.

### 4.3. The Improvement of Cardiac Metabolism and Calcium Homeostasis May Benefit DCM

Cardiac metabolic abnormalities are broadly recognized to increase various death risks. In diabetic heart, glucose oxidation was significantly decreased while the intake of fatty acids and its oxidation rate were further elevated. Increased dependence on fatty acids to generate energy may predispose the diabetic heart to the endoplasmic reticulum, oxidative stress, stress, and ischemic damage. Accumulation of intracellular toxic lipid metabolites gives rise to a great many cellular abnormalities resulting in cardiac dysfunction and cardiac remodeling [47]. Meanwhile, the abnormal mitochondrial membrane potential and permeability not only increased the production of reactive oxygen species but also impaired its elimination, causing accumulation of reactive oxygen species in diabetic heart. Excessive production of reactive oxygen species and loss of endothelial antioxidant barrier scales could lead to the production of oxidative stress. Additionally, calcium ion could enter the mitochondrial matrix mediated by a calcium uniporter complex in a sodium calcium exchanger manner, which helps to alleviate calcium overload. In our study, the GO analysis demonstrated that the DEGs were primarily enriched in fatty acid metabolism, mitochondrial membrane potential, mitochondrial uncoupling, glucose transporter, calcium ion binding, and lipid intake.

### 4.4. SOCS3 May Act as a Novel Therapeutic Target in DCM

The role of the IL-6/STAT3/SOCS3 signaling pathway in tumors has been well evaluated. IL6 stimulates survival, proliferation, and progression to cancer of intestinal epithelial cells via activation of signal transducers and activators of transcription 3 (STAT3), eventually inducing the expression of SCOS3 [47]. Meanwhile, SOCS3 is an important inhibitory factor of STAT3, which could block the phosphorylation of STAT3 and negatively regulate IL-6/STAT3 signaling [25]. Under normal circumstances, the activation of STAT3 is transitory and speedy, while STAT3 could be constitutively activated under pathologic status, which is attributed to the absence or downregulation of SOCS3 [48]. Currently, the role of SCOS3 in DCM remains unclear. We found that both IL6 and SOCS3 acted as hub genes in our study. Therefore, we put forward a hypothesis that IL6 trans-signaling may also activate STAT3/SOCS3, thus promoting the development of DCM. As expected, Western blotting showed that the protein expression of SOCS3 was significantly lower in the DCM group than that in the control group while the phosphorylation of STAT3 was significantly higher in the DCM group, indicating the inhibitory effects of SOCS3 on the phosphorylation of STAT3. To obtain more accurate results, further studies should be performed to explore the accurate mechanisms of the IL-6/STAT3/SOCS3 signaling pathway in DCM.

## 5. Conclusion

Conclusively, using a series of bioinformatics analysis, we give a comprehensive and novel illustration of gene expression profiles to identify DEGs expressing in myocardial tissue, which may play critical roles in the occurrence and development in patients with DCM. Genes and pathways implicated with inflammation, immune, and metabolism were significantly altered in DCM. Notably, IL6 may act as a much more important role in the development of DCM beyond our expectation. Additionally, targeting the IL-6/STAT3/SOCS3 signaling pathway is a promising strategy to treat DCM. These findings will greatly contribute to unveiling the molecule mechanisms of DCM. To allow these biomakers and targets to be used more routinely in clinic, further investigations into the correlation of plasma proteins as well as metabolites and these dysregulated genes should be performed.

## Figures and Tables

**Table 1:** The top 5 upregulated and downregulated differentially expressed genes in patients with diabetic cardiomyopathy.

DEGs	Gene title	Gene symbol	LogFC	Biological function
Upregulated	Natriuretic peptide A	NPPA	3.53	Extracellular fluid volume and electrolyte homeostasis
	Secreted frizzled related protein 4	SFRP4	2.71	Soluble modulators of Wnt signaling
	Desmocollin 1	DSC1	2.44	Calcium-dependent glycoprotein
	Nebulin	NEB	2.4	Cytoskeleton
	Frizzled-related protein	FRZB	2.36	Soluble modulators of Wnt signaling

Downregulated	Serpin family E member 1	SERPINE1	−2.48	Inhibitor of fibrinolysis
	Serpin family A member 3	SERPINA3	−2.44	Anti-inflammatory and antioxidant effects
	Ankyrin repeat domain 2	ANKRD2	−2.16	Modulator of NF-*κ*B-mediated inflammatory
	X-ray repair cross-complementing 4	XRCC4	−2.05	DNA repair
	S100 calcium-binding protein A8	S100A8	−1.98	Regulating inflammation and oxidative stress, activatingTLR4 signaling

**Table 2:** Gene ontology analysis of differentially expressed genes in patients with diabetic cardiomyopathy.

Expression	Category	Term	Count	%
Upregulated	GOTERM_BP_DIRECT	GO:0035589~G-protein-coupled purinergic nucleotide receptor signaling pathway	9	0.05
	GOTERM_BP_DIRECT	GO:0007155~fatty acid metabolism	8	0.05
	GOTERM_BP_DIRECT	GO:0001501~mitochondrial membrane potential	6	0.04
	GOTERM_BP_DIRECT	GO:0030198~extracellular matrix organization	6	0.04
	GOTERM_BP_DIRECT	GO:0007409~mitochondrial permeability transition	5	0.03
	GOTERM_CC_DIRECT	GO:0016021~integral component of membrane	42	0.25
	GOTERM_CC_DIRECT	GO:0005886~plasma membrane	37	0.22
	GOTERM_CC_DIRECT	GO:0070062~extracellular exosome	29	0.17
	GOTERM_CC_DIRECT	GO:0005615~extracellular space	22	0.13
	GOTERM_CC_DIRECT	GO:0005576~extracellular region	20	0.11
	GOTERM_MF_DIRECT	GO:0008270~glucose transporter	13	0.08
	GOTERM_MF_DIRECT	GO:0005509~calcium ion binding	11	0.07
	GOTERM_MF_DIRECT	GO:0008201~heparin binding	7	0.05
	GOTERM_MF_DIRECT	GO:0005518~collagen binding	5	0.03
	GOTERM_MF_DIRECT	GO:0050661~NADP binding	4	0.03

Downregulated	GOTERM_BP_DIRECT	GO:0006954~inflammatory response	11	0.07
	GOTERM_BP_DIRECT	GO:0008284~lipid intake	9	0.06
	GOTERM_BP_DIRECT	GO:0042493~response to drug	6	0.04
	GOTERM_BP_DIRECT	GO:0006955~immune response	6	0.04
	GOTERM_BP_DIRECT	GO:0002576~platelet degranulation	5	0.04
	GOTERM_CC_DIRECT	GO:0005886~plasma membrane	31	0.21
	GOTERM_CC_DIRECT	GO:0005615~extracellular space	23	0.15
	GOTERM_CC_DIRECT	GO:0005576~extracellular region	21	0.14
	GOTERM_CC_DIRECT	GO:0070062~extracellular exosome	21	0.14
	GOTERM_CC_DIRECT	GO:0005789~endoplasmic reticulum membrane	13	0.09
	GOTERM_MF_DIRECT	GO:0005515~protein binding	58	0.39
	GOTERM_MF_DIRECT	GO:0008083~mitochondrial uncoupling	5	0.03
	GOTERM_MF_DIRECT	GO:0005125~cytokine activity	5	0.03
	GOTERM_MF_DIRECT	GO:0003779~actin binding	5	0.03
	GOTERM_MF_DIRECT	GO:0016791~phosphatase activity	3	0.03

GO: gene ontology.

**Table 3:** KEGG pathway analysis of differentially expressed genes in patients with diabetic cardiomyopathy.

Category	Term	Count	%	*P* value	Genes
Downregulated DEGs	hsa04151:PI3K-Akt signaling pathway	8	0.05	0.01	FGF18, CDKN1A, IL6, FGF7, TNC, LAMC2, MYC, and SPP1
	hsa04010:MAPK signaling pathway	6	0.04	0.03	DUSP5, FGF18, FGF7, MAP2K3, FLNC, and MYC
	hsa04066:HIF-1 signaling pathway	4	0.02	0.03	CDKN1A, IL6, and TFRC
	hsa04668:TNF signaling pathway	4	0.02	0.04	IL6, SOCS3, MAP2K3, and SELE
	hsa04620:Toll-like receptor signaling pathway	4	0.02	0.04	IL6, LY96, MAP2K3, and SPP1

Upregulated DEGs	hsa00982:Drug metabolism-cytochrome P450	5	0.03	0.001	FMO4, FMO2, FMO3, ADH1B, and UGT2B4

KEGG: Kyoto Encyclopedia of Genes and Genomes; FDR: false discovery rate.

**Table 4:** Top 15 hub genes with higher degree of connectivity.

Gene	Degree of connectivity	*P* value
IL6	29	5.48*E* − 04
MYC	17	3.99*E* − 03
ACTA2	14	9.78*E* − 06
SERPINE1	14	1.16*E* − 02
ASPN	12	9.15*E* − 03
SPP1	11	4.04*E* − 02
KIT	11	4.54*E* − 03
TFRC	9	3.34*E* − 04
FMOD	9	3.42*E* − 04
PDE5A	9	2.96*E* − 04
MYH6	8	1.55*E* − 03
FPR1	8	1.71*E* − 06
C3	7	1.18*E* − 02
CDKN1A	7	3.94*E* − 04
SOCS3	7	1.02*E* − 03

**Table 5:** Gene-specific primers used in quantitative real-time PCR.

Species	Genes		Sequences
Mouse	GAPDH	Forward	5′-ACTCCACTCACGGCAAATTC-3′
		Reverse	5′-TCTCCATGGTGGTGAAGACA-3′

Mouse	NPPA	Forward	5′-CCCTCCGATAGATCTGCCCT-3′
		Reverse	5′-GTCAATCCTACCCCCGAAGC-3′

Mouse	SFRP4	Forward	5′-AAAAGCCGTCCAGAGGAGTG-3′
		Reverse	5′-GAGGGACTTGTGTTCGAGGG-3′

Mouse	DSC31	Forward	5′-GATCAGGCCAGTGGAAATGT-3′
		Reverse	5′-GTGTGTTTCGTGCAACCATC-3′

Mouse	NEB	Forward	5′-ATCCTGTCCAAACTAAGGCTCG-3′
		Reverse	5′-ACCTCTTTAGCATAGTAGTCCGC-3′

Mouse	SERPINE1	Forward	5′-GGGTTCACTTTACCCCTCCG-3′
		Reverse	5′-TAGGGCAGTTCCACAACGTC-3′

Mouse	SERPINA3	Forward	5′-TGACCTTTCTCAGCACGACC-3′
		Reverse	5′-AATAGGGGAGGATGGGAGCA-3′

Mouse	ANKRD2	Forward	5′-TTGCCCAGGAGGAAGAGACT-3′
		Reverse	5′-TGTCTCTCACGTTGGTGTCG-3′

Mouse	XRCC4	Forward	5′-TTGGGCGCATCGGTTTATCT-3′
		Reverse	5′-ACCAGTGCCTTTCTCAGCTC-3′

Mouse	S100A8	Forward	5′-TTCGTGACAATGCCGTCTGA-3′
		Reverse	5′-GGCCAGAAGCTCTGCTACTC-3′

## Data Availability

The data used to support the findings of this study are available from the corresponding author upon request.

## Supplementary Materials

Supplementary 1 Supplemental Table S1: gene-specific primers used in quantitative real-time PCR.

Supplementary 2 Figure S1: the construction of DCM in db/db mice. (A) Representative echocardiographic images in the control group and DCM group. (B) Representative images of the H&E staining and PSR staining in the indicated group.

Supplementary 3 Figure S2: the validation of top 5 upregulated and top 5 downregulated DEGs in vitro. (A) The mRNA expression of NPPA, SFRP4, DSC31, NEB, and FRZB in cardiomyocytes. (B) The mRNA expression of SERPINE1, SERPINA3, ANKRD2, ANKRD2, and S100A8 in cardiomyocytes. (C) The mRNA expression of NPPA, SFRP4, DSC31, NEB, and FRZB in cardiac fibroblasts. (D) The mRNA expression of SERPINE1, SERPINA3, ANKRD2, ANKRD2, and S100A8 in cardiac fibroblasts. ^∗^ *P* < 0.05 versus normal group.
