# Supplementary material for: Identification of Core Gene Biomarkers in Patients with Diabetic Cardiomyopathy
Source: Dis Markers. 2018 Dec 19;2018:6025061. doi: 10.1155/2018/6025061 (PMC6313979; doi:10.1155/2018/6025061)
Supplement: Supplementary 1 — Supplemental Table S1: gene-specific primers used in quantitative real-time PCR. [file 6025061.f1.docx]

**Supplemental data**

| Genes |  | Sequences |
| --- | --- | --- |
| GAPDH | Forward  Reverse | 5＇-ACTCCACTCACGGCAAATTC- 3＇  5＇-TCTCCATGGTGGTGAAGACA- 3＇ |
| NPPA | Forward  Reverse | 5＇-CCCTCCGATAGATCTGCCCT- 3＇  5＇-GTCAATCCTACCCCCGAAGC- 3＇ |
| SFRP4 | Forward  Reverse | 5＇-AAAAGCCGTCCAGAGGAGTG- 3＇  5＇-GAGGGACTTGTGTTCGAGGG- 3＇ |
| DSC31 | Forward  Reverse | 5＇-GATCAGGCCAGTGGAAATGT- 3＇  5＇-GTGTGTTTCGTGCAACCATC- 3＇ |
| NEB | Forward  Reverse | 5＇-ATCCTGTCCAAACTAAGGCTCG- 3＇  5＇-ACCTCTTTAGCATAGTAGTCCGC- 3＇ |
| SERPINE1 | Forward  Reverse | 5＇-GGGTTCACTTTACCCCTCCG- 3＇  5＇-TAGGGCAGTTCCACAACGTC- 3＇ |
| SERPINA3 | Forward  Reverse | 5＇-TGACCTTTCTCAGCACGACC- 3＇  5＇-AATAGGGGAGGATGGGAGCA- 3＇ |
| ANKRD2 | Forward  Reverse | 5＇-TTGCCCAGGAGGAAGAGACT- 3＇  5＇-TGTCTCTCACGTTGGTGTCG- 3＇ |
| XRCC4 | Forward  Reverse | 5＇-TTGGGCGCATCGGTTTATCT- 3＇  5＇-ACCAGTGCCTTTCTCAGCTC- 3＇ |
| S100A8 | Forward  Reverse | 5＇-TTCGTGACAATGCCGTCTGA- 3＇  5＇-GGCCAGAAGCTCTGCTACTC- 3＇ |

**Supplemental Table S1.** Gene-specific primers used in quantitative real-time PCR

**Figure legends**

**Figure S1.** The construction of DCM in db/db mice. (A) Representative echocardiographic images in control group and DCM group. (B) Representative images of the H&E staining and PSR staining in the indicated group.

**Figure S2.** The validation of top5 upregulated and top5 downregulated DEGs in vitro. (A) The mRNA expression of NPPA, SFRP4, DSC31, NEB and FRZB in cardiomyocytes. (B) The mRNA expression of SERPINE1, SERPINA3, ANKRD2, ANKRD2 and S100A8 in cardiomyocytes.(C) The mRNA expression of NPPA, SFRP4, DSC31, NEB and FRZB in cardiac fibroblasts.(D)The mRNA expression of SERPINE1, SERPINA3, ANKRD2, ANKRD2 and S100A8 in cardiac fibroblasts.* P < 0.05 versus normal group.
