# Supplementary figures and images for: Identification of Core Gene Biomarkers in Patients with Diabetic Cardiomyopathy
Source: Dis Markers. 2018 Dec 19;2018:6025061. doi: 10.1155/2018/6025061 (PMC6313979; doi:10.1155/2018/6025061)

Figure S1

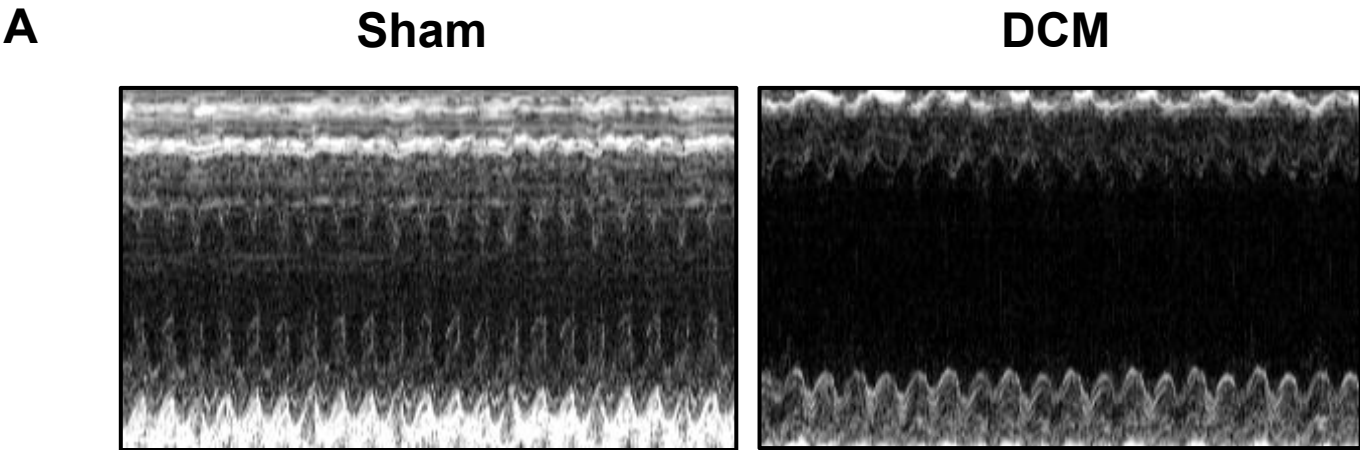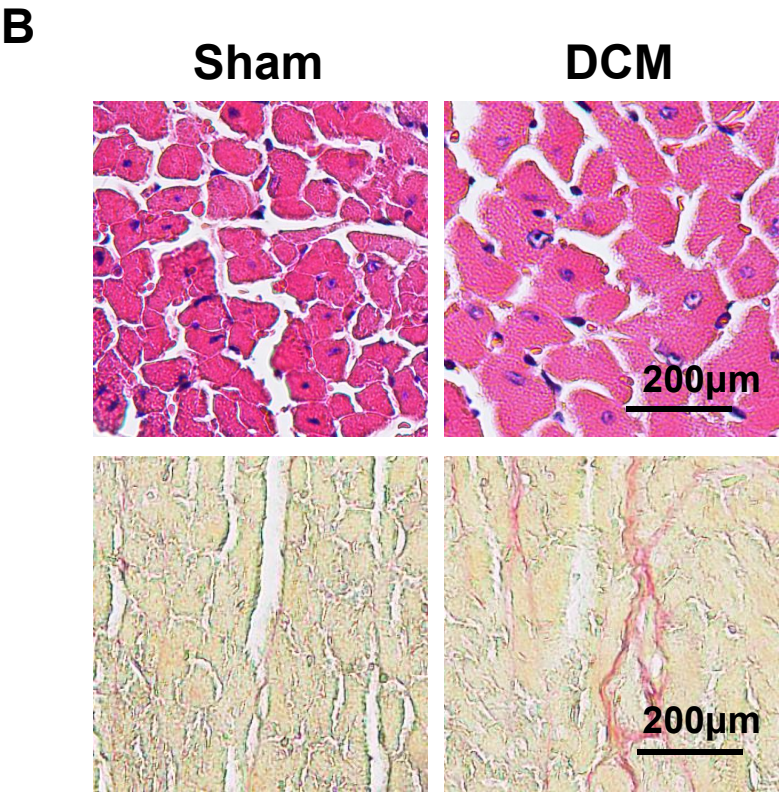

Supplement: Supplementary 2 — Figure S1: the construction of DCM in db/db mice. (A) Representative echocardiographic images in the control group and DCM group. (B) Representative images of the H&E staining and PSR staining in the indicated group. [file 6025061.f2.pdf]

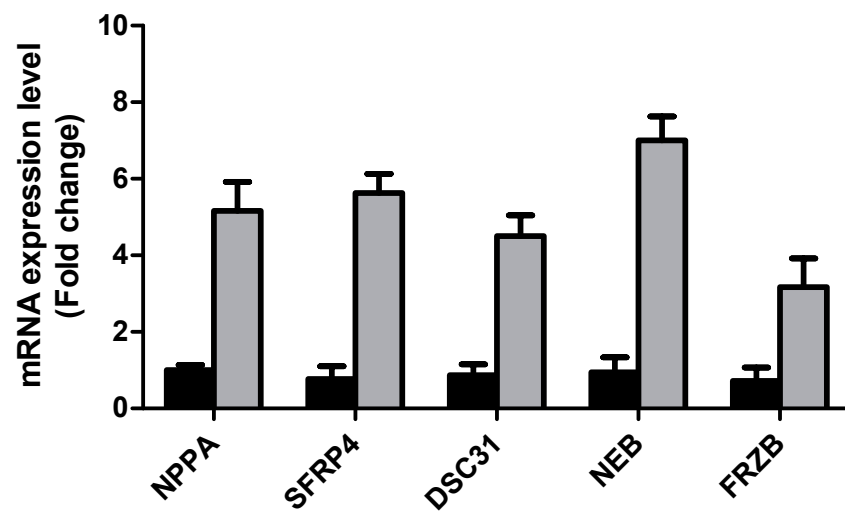

Supplement: Supplementary 3 — Figure S2: the validation of top 5 upregulated and top 5 downregulated DEGs in vitro. (A) The mRNA expression of NPPA, SFRP4, DSC31, NEB, and FRZB in cardiomyocytes. (B) The mRNA expression of SERPINE1, SERPINA3, ANKRD2, ANKRD2, and S100A8 in cardiomyocytes. (C) The mRNA expression of NPPA, SFRP4, DSC31, NEB, and FRZB in cardiac fibroblasts. (D) The mRNA expression of SERPINE1, SERPINA3, ANKRD2, ANKRD2, and S100A8 in cardiac fibroblasts. ∗ P < 0.05 versus normal group. [file 6025061.f3.zip › Figure S2/Figure S2A_DM_2469719.pdf]

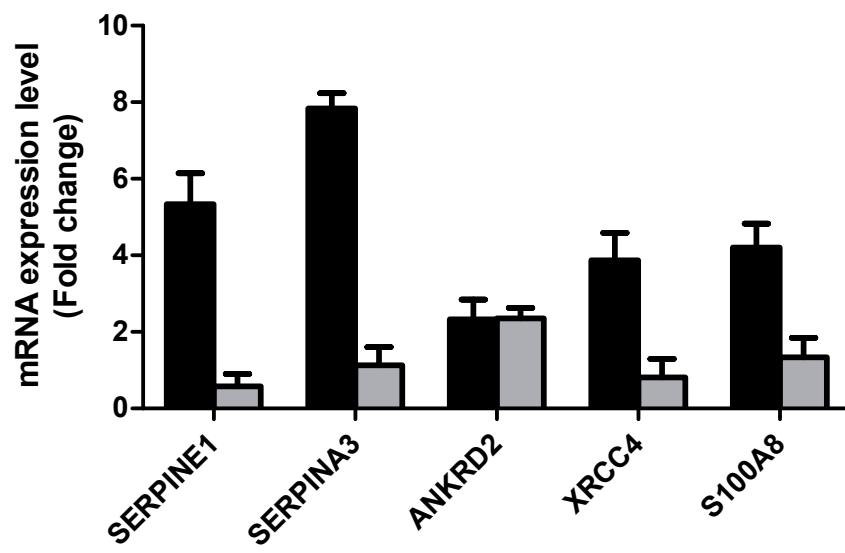

Supplement: Supplementary 3 — Figure S2: the validation of top 5 upregulated and top 5 downregulated DEGs in vitro. (A) The mRNA expression of NPPA, SFRP4, DSC31, NEB, and FRZB in cardiomyocytes. (B) The mRNA expression of SERPINE1, SERPINA3, ANKRD2, ANKRD2, and S100A8 in cardiomyocytes. (C) The mRNA expression of NPPA, SFRP4, DSC31, NEB, and FRZB in cardiac fibroblasts. (D) The mRNA expression of SERPINE1, SERPINA3, ANKRD2, ANKRD2, and S100A8 in cardiac fibroblasts. ∗ P < 0.05 versus normal group. [file 6025061.f3.zip › Figure S2/Figure S2B_DM_2469720.pdf]

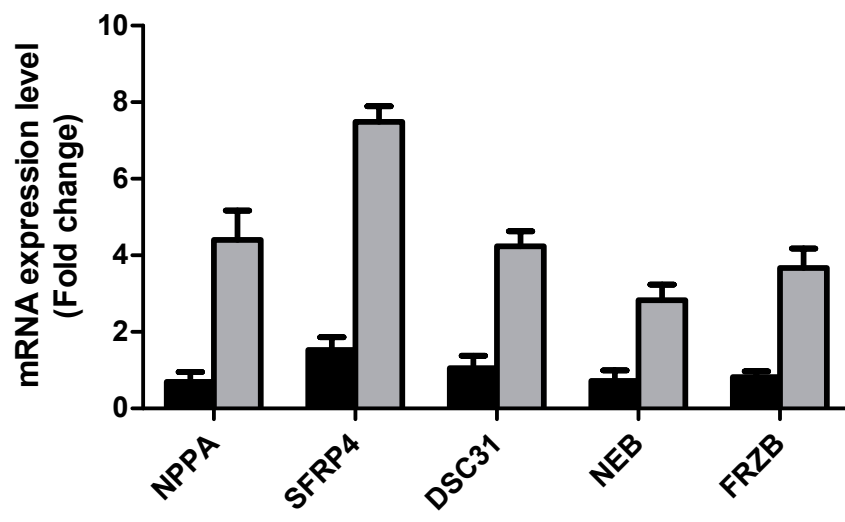

Supplement: Supplementary 3 — Figure S2: the validation of top 5 upregulated and top 5 downregulated DEGs in vitro. (A) The mRNA expression of NPPA, SFRP4, DSC31, NEB, and FRZB in cardiomyocytes. (B) The mRNA expression of SERPINE1, SERPINA3, ANKRD2, ANKRD2, and S100A8 in cardiomyocytes. (C) The mRNA expression of NPPA, SFRP4, DSC31, NEB, and FRZB in cardiac fibroblasts. (D) The mRNA expression of SERPINE1, SERPINA3, ANKRD2, ANKRD2, and S100A8 in cardiac fibroblasts. ∗ P < 0.05 versus normal group. [file 6025061.f3.zip › Figure S2/Figure S2C_DM_2469721.pdf]

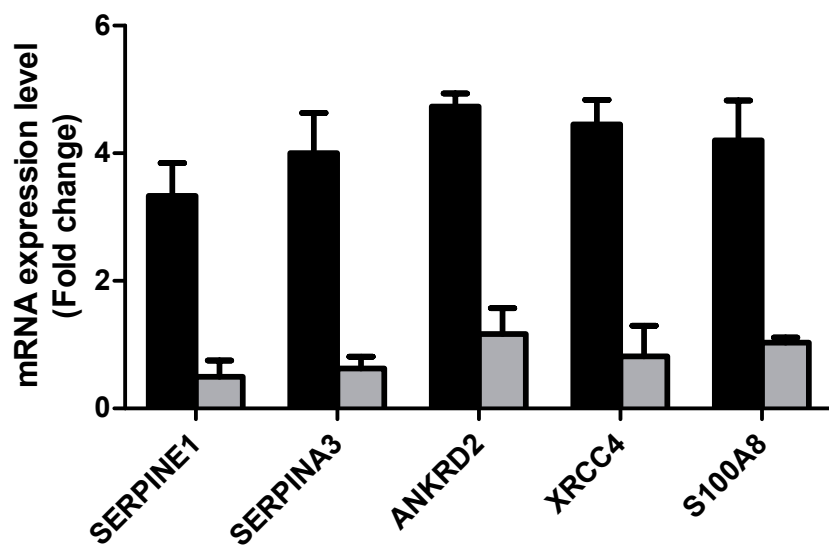

Supplement: Supplementary 3 — Figure S2: the validation of top 5 upregulated and top 5 downregulated DEGs in vitro. (A) The mRNA expression of NPPA, SFRP4, DSC31, NEB, and FRZB in cardiomyocytes. (B) The mRNA expression of SERPINE1, SERPINA3, ANKRD2, ANKRD2, and S100A8 in cardiomyocytes. (C) The mRNA expression of NPPA, SFRP4, DSC31, NEB, and FRZB in cardiac fibroblasts. (D) The mRNA expression of SERPINE1, SERPINA3, ANKRD2, ANKRD2, and S100A8 in cardiac fibroblasts. ∗ P < 0.05 versus normal group. [file 6025061.f3.zip › Figure S2/Figure S2D_DM_2469722.pdf]
